# 

**Published:** 1916-02

**Authors:** 


### CONTENTS, FEBRUARY, 1916—VOLUME XXXI, NO. 8.

### ORIGINAL ARTICLES.

Involution Melancholia. By J. A. McIntosh, San Antonio, Texas . . 307
Diphtheria. By Dr. M. L. Wilbanks, Greenville, Texas . . . . . . . . . . 312
Abstract of Clinical Lecture in the Long Island Hospital on the Diagnosis of Syphilis. By Henry H. Morton, M. D . . . . . . . . . 318

### DEPARTMENT OF EUGENICS.

Extracts from an address delivered in Chicago December 10, 1915, before the Eugenics Educational Society. By Casper L. Redfield . . . . . . . . . 321
Is Contraception Injurious to Health? . . . . . . . . . . 324

### EDITORIAL DEPARTMENT.

The Doctor . . . . . . . . . . 330
Exterminate the Pests . . . . . . . . . . 331
New Diseases . . . . . . . . . . 332
Must Reorganize . . . . . . . . . . 332
Pellagra Increasing . . . . . . . . . . 333
Prenatal Care of Infants . . . . . . . . . . 333
Health Sunday . . . . . . . . . . 333
Safeguard Ethics . . . . . . . . . . 334
Rats and Mice . . . . . . . . . . 334
Items of General Interest . . . . . . . . . . 335
Personals . . . . . . . . . . 338
Deaths . . . . . . . . . . 342
Book Reviews . . . . . . . . . . 344
Helpful Hints for the Busy Doctor . . . . . . . . . . 347

